# Anxiety, Depression, Social Support, Needs, and Concerns of Frontline Nurses during COVID-19 Peak Infection Period: A Cross-Sectional Multicenter Study

**DOI:** 10.1155/2024/6007430

**Published:** 2024-06-28

**Authors:** Liuyi Zhang, Kemei Zhang, Li Tong, Yafen Guo, Jinhua Shen, Xue-qing Zhang, Pan Yang

**Affiliations:** ^1^ Department of Nursing Medical College of Hunan Normal University, Changsha 410013, Hunan, China; ^2^ Ophthalmology Ward II Changde Hospital Xiangya School of Medicine Central South University (The First People's Hospital of Changde City), Changde 415000, Hunan, China; ^3^ Department of Nursing Changde Hospital Xiangya School of Medicine Central South University (The First People's Hospital of Changde City), Changde 415000, Hunan, China

## Abstract

**Background:**

The worldwide pandemic of COVID-19 had put enormous pressure on frontline healthcare workers. In December 2022, China released its “10 new measures,” signaling the end of the “dynamic zero COVID-19 strategy.” This triggered a period of peak infection, which shocked China's healthcare system and affected the mental health of nurses.

**Objective:**

To explore the anxiety, depression, and social support levels of frontline nurses during the peak period of COVID-19 infection and to identify the main needs and concerns of nurses during this period.

**Design:**

A multicenter cross-sectional study. *Settings*. 18 hospitals of different grades in three cities in Hunan Province from December 21, 2022, to January 10, 2023, the peak period of COVID-19 infection. *Participants*. A total of 4,160 nurses completed the survey.

**Methods:**

The questionnaire included general information, symptoms, the preparation for nurses, GAD-7, PHQ-9, PSSS, and two open-ended questions investigating nurses' needs and concerns. General linear models were used to analyze the factors influencing anxiety. Hospital preparation for nurses, nurses' needs, and nurses' concerns were categorized and subjected to frequency counts.

**Results:**

The median (P25, P75) scores for anxiety and depression among nurses were 7.00 (3, 12) and 8 (3, 12), respectively. Type of hospital, professional title, family structure, isolation staff lounge preparation, ibuprofen preparation, health status of parents, fever, chest distress, dyspnea, cough, insufficient protective equipment, number of children, and PSSS others were the influencing factors of GAD grades. The top 3 needs were free drugs and treatments (78.71%), shift breaks and paid leave (77.66%), and understanding and supports from hospitals and families (75.99%). The first three concerns were the fear of spreading the disease to family members (83.89%), the after-effects of infection (65.67%), and cross-infection with colleagues and patients (61.70%).

**Conclusions:**

Nurses' anxiety was more severe during peak infection period. Overloaded work schedules and insufficient sleep became a common situation. Worries about family members became the main concern of nurses. Managers should make contingency plans for public health emergencies and provide frontline nurses with protective equipment, stress-relieving measures, and a rotation system. More importantly, they should pay attention to the needs of nurses' family members and provide medical and care support. The media should also explain how hospitals operate and carry out their duties during these extraordinary times in order for the public to comprehend the condition of frontline epidemic fighters.

## 1. Introduction

The COVID-19 worldwide epidemic had lasted for three years since the outbreak was reported in central China's Wuhan city in December 2019. Because of the differences in social cultural, political, and economic contexts in different countries, there has been no consensus prevention policy about the public health strategy for COVID-19. Before December 7, 2022, the Chinese government had adopted a “dynamic zero COVID-19 strategy” to control the pandemic, in which restrictive measures were initiated and maintained until there were no documented COVID-19 cases in a particular geographic location [1]. In this period, facing with COVID-19 epidemic such as a catastrophic health emergency, the mental health status of frontline medical staff deserves attention [2]. Studies [3, 4] have shown that the prevalence of anxiety, depression, and sleep disorders among Chinese medical professionals during the epidemic ranged from 44.6% to 81.36%, 45.76% to 50.4%, and 34.0% to 97.88%, respectively. To deal with the situation, a national guideline for psychological crisis intervention for COVID-19 was released by the Chinese National Health Commission, marking the beginning of China's efforts to offer medical professionals' full psychological protection [5].

However, on December 7, 2022, the China National Health Commission issued “10 new measures” to further optimize the implementation of prevention and control of the COVID-19 epidemic [6]. The “10 new measures” required scientific and precise delineation of risk areas to minimize the impact of the epidemic on economic and social development, which indicated that China had relaxed its strict COVID-19 prevention and control measures. Thus, widespread infection—including among frontline medical personnel—was unavoidable. There were 13.983 million medical professionals working in China at that time, including 5.018 million registered nurses and 4.287 million practicing physicians and physician assistants [7]. Therefore, there would be more risk and strain on healthcare facilities and medical staff as a result of the new policy shift. Considering that previous research has found that during the COVID-19 pandemic, medical staff self-reported high rates of anxiety and depression symptoms, and psychological interventions for those at high risk of psychological disorders should be integrated into the work plan to fight the pandemic [8].

Most COVID-19 cases were mild. After infection, some people might not even exhibit any clinical symptoms [9]. On the other hand, a few people could develop a severe COVID-19 case. Age, gender, obesity, smoking, and comorbidity chronic diseases like hypertension, type 2 diabetes mellitus, and others were key risk factors [10, 11]. Yet, the population's immunity had further decreased due to the “immunization debt” caused by mask-wearing and reduced sociability policies that China had imposed over the past three years [12], as well as the country's rapidly aging population, which some had dubbed an “aging tsunami” [13]. These factors, in addition to China's huge population, fostered tremendous challenges for healthcare.

According to data released on the official website of the China Center for Disease Control and Prevention (CDC) [14], the number of infections peaked around December 22, 2022, with a maximum of over 7 million new infections per day. The number of fever outpatient (clinic) visits nationwide (excluding Hong Kong, Macao, and Taiwan) peaked on December 23, 2022, at 2.867 million visits. The number of severely ill patients among newly coronavirus-infected patients in hospitals nationwide increased by nearly 10,000 per day from December 27, 2022, to January 3, 2023, and then decreased significantly on January 4 before reaching a peak of 128,000 on January 5th. The number of deaths from new coronavirus infections in hospitals reached a daily peak of 4,273 on January 4th. Such a large number of patients was undoubtedly an overload for healthcare workers, especially when they themselves were infected. The impact of such a large-scale infection in a short period of time on China's healthcare system was enormous and undoubtedly posed a huge challenge to the physical and psychological limits of medical workers [15].

This daunting task might cause not only significant psychological stress but also a shift in working state [16], especially in specific COVID-19 units and emergency departments [17]. Burnout, intentions to leave [18], and compassion fatigue [19] would emerge, especially in the absence of psychological counseling and professional support. Furthermore, sleep deprivation caused by heavy work might impair emotional awareness, resulting in decreased levels of empathy [20]. The situation seems even less promising in Chinese collectivist cultures. Collectivism requires a greater focus on the feelings of others and therefore risks neglecting one's own requirements [21]. Therefore, we need to focus on and enhance support for healthcare workers so that they can become aware of their emotions and can help them remain productive and focused during stressful events of a pandemic [22].

Nevertheless, studies on the psychological state and social support of medical staff during the COVID-19 peak infection period remained mostly unexplored, particularly in light of the large number of infections with COVID-19 and a shortage of medical staff in China. Therefore, this study aimed to investigate the anxiety, depression, stressors, social support, and needs of frontline nurses during COVID-19 peak infection period in China. This will provide the groundwork for healthcare workers' social support and psychological intervention during an epidemic.

## 2. Methods

### 2.1. Study Design

A multicenter cross-sectional survey was conducted in 18 hospitals in three cities: Changde, Changsha, and Chenzhou in northern, central, and southern Hunan Province in southcentral China, from December 21, 2022, to January 10, 2023, the COVID-19 peak infection period after the release of “10 new measures” by the Chinese government.

### 2.2. Settings

**Figure 1:**
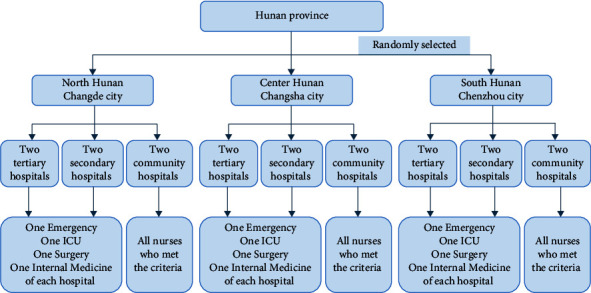
Flowchart of selecting settings.

Randomized stratified sampling was used to select hospitals. First, three cities from the north, center, and south of Hunan Province were chosen. Then, six hospitals or community health centers were randomly selected from each city, respectively. Among them, two were tertiary, two were secondary, and two were community hospitals. In all, eighteen hospitals in the three cities were selected. Following that, one emergency room, ICU, surgery, and medical department were chosen at random from each tertiary and secondary hospital. The survey was distributed to all of the nurses in the chosen department who met the criteria. Since there were not enough community nurses, the study included all of those who met the criteria. The specific process is shown in Figure 1.

### 2.3. Participants and Sample Size

Nurses were included in the study if they (1) were registered nurses; (2) were on duty; (3) did not have serious psychiatric disorders, such as schizophrenia and depression; and (4) gave informed consent to participate in this study. The exclusion criteria were (1) being on vacation or undergoing a training course in another hospital during the data collection period and (2) recently experiencing a major psychiatric event, such as divorce and death of a family member.

The sample size was calculated using the method of 10 EPV (events per variable) [23]. A total of 26 variables were analyzed in the influential factors of anxiety in this study, so at least 260 nurses with severe anxiety were needed. According to the references, the prevalence of severe anxiety among nurses during the COVID-19 epidemic was 3.1% [24], 4.5% [25], and 17.2% [26], respectively. In addition, we used a mean value of 8%. Therefore, at least (260/0.08) = 3250 nurses need to be surveyed. Assuming a 20% of attrition, we will recruit a minimum of 4063 participants.

### 2.4. Outcome Measures

The questionnaire included seven parts: demographic information, the preparation of the medical staff before the implementation of “10 new measures,” the patient health questionnaire 9, the general anxiety disorder 7, the perceived social support scale, the needs of nurses, and the concerns of nurses during COVID-19 peak infection period.

#### 2.4.1. Sociodemographics and Symptoms of Infection

This part collected the nurses' gender, age, hospital classification, hospital category, professional title, marital status, education, vaccination, family structure, health status of parents, number of children, and whether nurses showed symptoms of infection (e.g., fever, malaise, and respiratory distress).

#### 2.4.2. The Preparation for the Nurses

This part investigated what hospitals prepared for the nurses and their families, such as isolation staff lounge preparation, drugs preparation, children, and parents care preparation (e.g., medication emergency kits for the elderly and children) after the “10 new measures” implementation.

#### 2.4.3. General Anxiety Disorder 7 (GAD-7)

The GAD-7 is a seven-item self-report scale that assesses anxiety, which was developed by Spitzer et al. [27] and translated into Chinese by He et al. [28]. Each item is scored from 0 to 3, with a total score ranging from 0 to 21. Anxiety symptoms can be categorized into four degrees based on total scores [28, 29]: no anxiety (≤4 points), mild (5–9 points), moderate (10–14 points), and severe anxiety (≥15 points). The GAD-7 is a reasonably accurate screening tool for anxiety disorders and symptoms, according to numerous research. In the current study, Cronbach's alpha for the GAD-7 was 0.80.

#### 2.4.4. Patient Health Questionnaire 9 (PHQ-9)

The PHQ-9 is a nine-item self-report measure that was developed by Kroenke et al. to assess depression symptoms [30]. The score of each item ranges from 0 to 3, and the total score ranges from 0 to 27. Symptom severity according to the total score can be divided into four grades: mild (5–9 points), moderate (10–14 points), moderately severe (15–19 points), and severe depression (≥20 points) [31]. The scale was shown to have good reliability and validity. The Chinese version translated by Chen et al. [32] was used in this study. Cronbach's alpha for the scale in the present study was 0.84.

#### 2.4.5. Perceived Social Support Scale (PSSS)

Zimet et al. [33] created the 12-item self-report measure that evaluates how much social support one receives from friends, family, and significant others on a subjective level. Every item is rated using a 7-point Likert scale. Total scores of 12–36 indicate a low level of support, 37–60 suggest an intermediate level of support, and 61–84 show a high level of support. The Chinese version translated by Chou [34] was used in this study. Cronbach's alpha coefficient for the total PSS was 0.89 in this study.

#### 2.4.6. The Needs of Nurses

Nurses were asked to list up to five of their most pressing needs in response to an open-ended question to assess their circumstances while working on the front line of the epidemic fight.

#### 2.4.7. The Concerns of Nurses

This section was also an open-response section that asked the frontline nurses to provide up to 5 of their current top concerns.

### 2.5. Data Collection

The anonymous self-assessment questionnaire for the survey was prepared using an online crowdsourcing platform in mainland China (https://www.wjx.cn), and it was sent to nurses via the Internet. Each hospital had a dedicated trained researcher assigned to it for quality control, data collecting, and hospital-to-hospital communication.

### 2.6. Statistical Analysis

Statistical descriptions and analyses were conducted for quantitative information using SPSS 26.0 for Windows (SPSS, Chicago, IL, USA). Normally distributed measures were presented as Mean (SD), and nonnormally distributed information was presented as Median (P25, P75). A generalized linear model (GLM) was used to explore factors leading to anxiety. All analyses were performed using a two-sided test *P* < 0.05. Answers to open-response questions were categorized by researchers.

### 2.7. Ethics Approval and Consent

The study was approved by the Ethics Committee of the Hospital (No. 2023-005-01). A covering letter explaining the objectives of the research and data privacy policies was given with the questionnaires distributed to the nurses. It was emphasized that participation was entirely voluntary and that completing the questionnaire or not would not affect their work and life in any way. The informed consent page presented two options (yes/no). Only participants who chose yes were taken to the questionnaire page, and participants could quit the survey at any time. To ensure data protection and confidentiality, codes were used for each hospital and participants were completely anonymous.

## 3. Results

### 3.1. Characteristics, Anxiety, Depression, and Social Support of Nurses

A total of 4,379 nurses met the inclusion criteria, and finally, 4,160 nurses responded and completed the questionnaire from December 21, 2022, to January 10, 2023. After excluding invalid questionnaires (e.g., choosing the same answer for all questions, or illogical responses, such as age 120 years), 4,128 valid questionnaires were finally included. The response rate was 95.00%, and the validity rate was 99.23%. The median (P25, P75) scores for anxiety, depression, and perceived social support among nurses were 7.00 (3, 12), 8 (3, 12), and 63 (52, 72), respectively. Higher GAD scores were reported by nurses who were females, worked at general hospitals or tertiary-level hospitals, held midlevel job titles, were divorced, held a bachelor's degree, were unvaccinated, belonged to three-generation households, had two or more children, and both parents were in poor health, while lower PHQ scores were reported among nurses in primary or community hospitals, senior nurses, widowed nurses, nurses with doctoral degrees, nurses who had completed vaccinations, nurses who lived alone, nurses whose parents were both in good health, and nurses who had no children. Additionally, higher PSSS scores were noted for tertiary-level hospital nurses, general hospital nurses, nurses in senior positions, married nurses, nurses with a small family structure or couples living together, nurses with both parents in good health, and nurses with two children.

Demographic characteristics and the anxiety, depression, and perceived social support scores of nurses are shown in Table 1.

### 3.2. Nucleic Acid Test Results and Symptoms of Nurses

Nucleic acid tests were reported positively by 1532 (37.11%) nurses and negatively by 1338 (32.41%) nurses. Besides, 1258 nurses (30.47%) did not perform a nucleic acid test. The partitions of the chi-square method were used to compare each group's nurses' vaccination completion rates. A statistically significant difference in vaccine completion rates was identified between nurses with positive nucleic acid test results with symptoms versus those with negative test results and without any symptoms. The comparison of nucleic acid test findings with symptoms and vaccination implementation is shown in Table 2.

As for the specific symptoms, dyspnea was the most reported symptom, occurring in 89.07% of nurses. 90.75% (3746/4128) of the nurses reported at least one infection-related symptom, such as chest tightness and fever.  Table 3 shows the specific symptoms of nurses.

### 3.3. The Preparation for the Nurses

**Figure 2:**
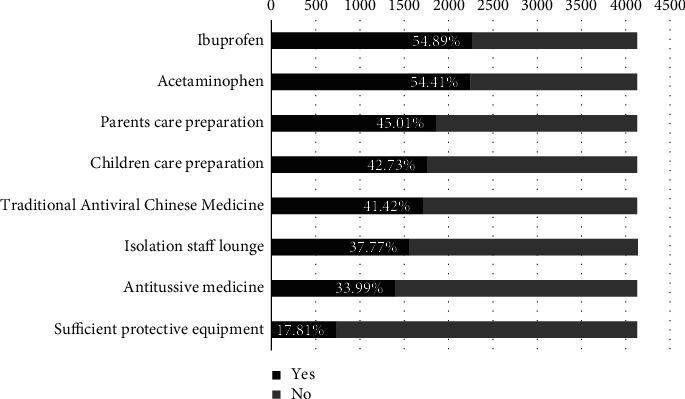
Hospital preparation for nurses before the peak infection.

Hospitals' preparations for healthcare workers following the adoption of the “10 new measures” are shown in Figure 2. The most prepared were ibuprofen and acetaminophen at 54.89% and 54.41%, respectively, while the least prepared were antitussive medicines and protective equipment, with only 33.99% and 17.81%, respectively.

### 3.4. The Generalized Linear Model of Anxiety

The GAD data were converted into hierarchical categorical variables (none, mild, moderate, and severe anxiety) due to the GAD scores' nonnormal distribution. Multivariate ordered logistic regression analyses were then carried out in a generalized linear model to examine the influence factors of GAD. GAD grades were analyzed as the dependent variable, meaningful demographics, hospital preparation for nurses, and whether nurses showed symptoms of infection were analyzed as independent variables. The results showed that type of hospital, professional title, family structure, isolation staff lounge preparation, ibuprofen preparation, health status of parents, fever, chest distress, dyspnea, cough, insufficient protective equipment, number of children, and PSSS others were the influencing factors of GAD grades. In contrast, gender, hospital grade, marital status, education level, and vaccination status were no longer statistically significant, see Table 4.

### 3.5. Nurses' Needs during COVID-19 Peak Infection Period

**Figure 3:**
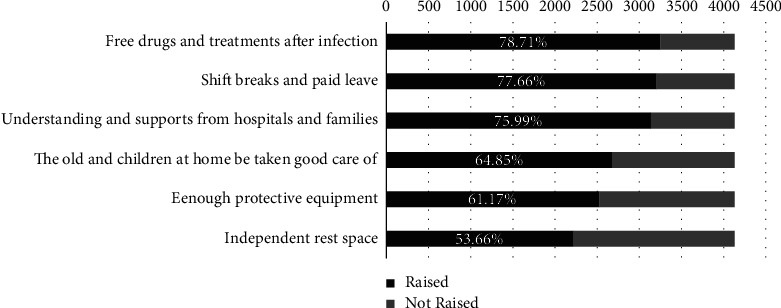
The major needs of nurses during COVID-19 peak infection period.

Figure 3 shows the major needs of nurses during COVID-19 peak infection period. The top three needs were free drugs and treatments (78.71%), shift breaks and paid leave (77.66%), and understanding and supports from hospitals and families (75.99%).

### 3.6. Nurses' Concerns during COVID-19 Peak Infection Period

**Figure 4:**
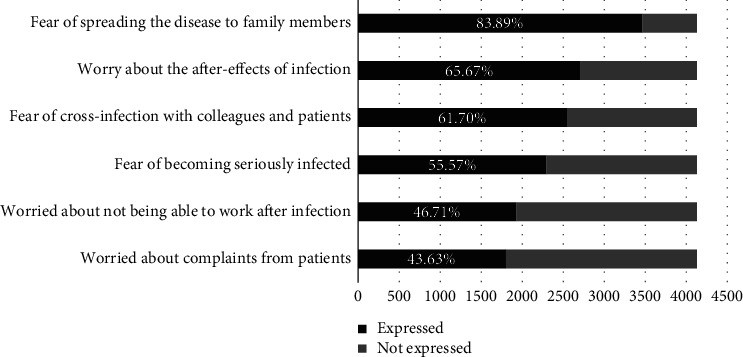
The major concerns of nurses during COVID-19 peak infection period.

The main worries that nurses had during the COVID-19 peak infection period are depicted in Figure 4. The leading three concerns were the fear of spreading the disease to family members (83.89%), worry about the after-effects of infection (65.67%), and the fear of cross-infection with colleagues and patients (61.70%).

## 4. Discussion

This study examined the impacts of the Chinese government's release of the “10 new measures” and the cancellation of the “dynamic zero COVID-19 strategy” on the mental health of Chinese healthcare professionals during the rapid peak infection of COVID-19. Understanding the psychological issues and needs of frontline nurses during peak infection periods could help in the development of stress-relieving solutions for healthcare professionals, as well as mitigation strategies for future infectious disease pandemics or catastrophic disasters.

### 4.1. Psychological Pressure on Medical Staff Was Greater during Peak Epidemics

Medical staff will exhibit psychological symptoms including mental health conditions like anxiety and depression when faced with an outbreak during an infectious disease pandemic [35–37]. Even worse, the rapid rise of COVID-19 infections in China after the new policy was announced put a tremendous deal of strain on the country's healthcare system and increased psychological stress on medical workers. The median anxiety score in this study was 7, which was higher than the scores of 6, 6, and 4 in Spain [38], Australia [39], and Korea [40]. The reasons may include the following: Firstly, it had to do with the virus's increased contagiousness following mutations [41] that resulted in a higher number of infections in a short period of time. Secondly, older people were more likely to have severe cases of COVID-19 [42], while the Chinese population is aging rapidly [43]. Thirdly, Chinese people value collectivism and the idea that other people's interests and safety should come first in dangerous circumstances [44]. Lots of nurses worked with illness at the expense of their own and their families' well-being because of the sense of dedication, which would exacerbate their emotions of shame and anxieties for family members [45, 46].

### 4.2. Factors Associated with Nurses' GAD Grades during COVID-19 Peak Infection Period

The results showed that having a junior professional title, working in a general hospital, living with parents, husband and children, having parents in poor health, having 2 or more children to care for, having a fever over 39°C after infection, and having symptoms of chest pain increased the risk of anxiety among medical staff. Usually, the higher the professional title of the medical staff and the more years they have worked, the better their coping skills and psychological profile, which to some extent reduces the risk of anxiety and depression. This is similar to the findings of previous studies [4], where midlevel technical titles were associated with experiencing severe depression, anxiety, and distress. Furthermore, given the lack of adequate personal protective equipment in most hospitals and the fact that the majority of nurses contract infections while on the job, nurses in administrative or senior roles were less likely to be exposed to confirmed patients and, consequently, experienced less psychological impact.

Interestingly, there were differences in anxiety levels among nurses with different vaccination completion statuses, but vaccination status was not statistically significant in the generalized linear model. However, the chi-square test revealed that vaccine completion status did correlate with nucleic acid test results and symptoms. The vaccination may have prevented infection in some nurses, but this group was so small—only 3.75% of nurses with negative nucleic acid tests, no symptoms, and vaccination completion—that the nurses were unsure they would not contract the infection even after receiving the vaccination, especially given the majority of the population was infected. Secondly, a sizable percentage of nurses (28.56%) exhibited symptoms despite having a negative nucleic acid test result. It could not be ruled out, nevertheless, that some of these nurses experienced symptoms unrelated to infection as a result of intense workloads and excessive psychological stress. However, the psychological effects of the vaccine were overshadowed by other factors since these nurses believed they were infected.

Our research also revealed a link between anxiety in nurses and COVID-19 infection symptoms as well as family members' health status. Previous research findings have demonstrated that direct contact with COVID-19 patients as frontline healthcare workers was an independent risk factor for all psychology symptoms [4]. It is conceivable that a significant proportion of healthcare workers would additionally unavoidably contract COVID-19 patients after the release of the new policy. As a result, physiological symptoms such as fever, chest tightness, dyspnea, and coughing that occur after infection aggravate nurses' anxiety. In addition, concerns about the health status of elderly parents also became an aggravating factor for the nurses' psychological burden. Because of the disease's dual effects of physical and psychological stress, frontline healthcare workers would be especially vulnerable to symptoms like anxiety, depression, insomnia, and distress, and their mental health required extra attention.

Interestingly, this study found that social support from the hospital affected nurses' anxiety levels, whereas support from family and friends did not. The possible reason for this is related to the collectivist culture mentioned above—nurses may feel guilty about their family and friends—so support from family friends did not reduce their anxiety. Compared to nurses working in general hospitals, nurses employed at specialized and community hospitals reported lower levels of anxiety. This is likely due to the fact that these institutions admitted fewer COVID-19 critically ill patients [47]. In addition, related research has shown that nurses working in COVID-19 facilities were 2.62 times more likely to have higher emotional eating behaviors compared to nurses in non-COVID-19 facilities [48]. This suggested that nurses in COVID-19 facilities may have higher levels of psychological stress and are more deserving of attention.

### 4.3. Worries about Family Members Became the Main Concerns of the Nurses

The results of the two open-ended questions on nurses' needs and concerns showed that worries about family members had become the nurses' main concern. 83.89% of the nurses expressed the fear of spreading diseases to their family members, while 64.85% of the nurses expressed the needs to take care of old people or children at home, which was consistent with the findings of Nashwan et al. [49]. Firstly, it has to do with the predominant family structure of small family and three-generation family in China [50]. The study's general data also revealed that the two types of families—small families, which consist of couples and children, and three-generation families, which consist of parents, couples, and children—accounted for the majority of the components, which were 33.19% and 28.08%, respectively. In the two types of families, nurses were expected to take on the role of primary caregiver for family members, with a focus on the elderly and children. Secondly, nurses may infect their families with the virus when they return home from work. That is why 53% of nurses cited the need for separate rest spaces—they did not want to spread the virus to their families. Therefore, managers can appropriately protect and comfort the family members of medical staff when making plans for the prevention and control of infectious diseases, which will lessen the stress of frontline medical professionals.

### 4.4. Overloaded Work Schedules and Lack of Rest Became a Common Situation

The findings showed that 76.31% of the participants had a fever of 39°C or above. Nurses with more severe symptoms really had to take time off from work, while nurses with less severe symptoms had to stay on the job and often even put in extra hours to make up for the personnel deficit caused by infection [51]. Therefore, in this study, many nurses raised the need for rest or paid leave. It was surprising to learn that 43.63% of the participants were worried about patient complaints. Studies do, in fact, indicate a link between higher complaint rates and a lack of human resources [52]. However, the majority of nurses were completing their work in a state of illness. Therefore, the media should publicize this more so that the public understands the working conditions of nurses and the high stress situations in which they work. Managers should provide more social support—it was also confirmed in this study that support from organizations (PSSS others) could significantly reduce nurses' anxiety state.

## 5. Limitations

The study had a few limitations. Firstly, asking nurses to fill out the questionnaire at a time when they were busy and under high stress may yield untrue results and add to their burden; secondly, as this study investigated the psychological state of nurses in the acute phase, depression was not discussed in depth—since the nurses were in a state of stress and showed more courage, while depressive states might continue to rise long after the stressful event is over [53], according to Selye's [54, 55] three-stage general adaptation syndrome (alarm, resistance, and exhaustion). Consequently, it is important to follow the psychological well-being of nurses and to implement interventions following the epidemic's peak; thirdly, this study was conducted in one province in China, which may have resulted in sampling bias due to differences between provinces or regions; fourthly, as had mentioned before, the study was conducted over a short period of time and lacked longitudinal follow-up. Due to the increasing severity of the situation, the mental health symptoms of healthcare workers may become more severe. Therefore, the long-term psychological impact of this population warrants further research.

## 6. Conclusions

This study investigated the psychological state of nurses at the peak of the COVID-19 outbreak in China after the abandonment of the “dynamic zero COVID-19 strategy.” Nurses' anxiety was more severe, with hospital type, family structure, protective preparation, medication preparation, symptoms, parental state of health, number of children, and social support being the main influencing factors. Most of nurses were infected during that period. Overloaded work schedules and lack of rest became a common situation. Worries about family members became the main concerns of the nurses.

## 7. Relevance to Clinical Practice and Education

In times of large-scale epidemics of infectious diseases, medical human resources are in short supply and medical staff are under greater stress. Managers should make contingency plans and consider the care and protection of medical staff's family members. The media should explain the operation and work of hospitals in extraordinary times so that the public understands the state of frontline epidemic fighters.

## Figures and Tables

**Table 1:** Demographics, anxiety, depression, and social support of nurses.

		Total (*n* = 4128)	GAD		PHQ		PSSS	
			Median (P25, P75)^*∗*^	Z/H(p)^§^	Median (P25, P75)^*∗*^	Z/H(p)^§^	Median (P25, P75)^*∗*^	Z/H(p)^§^
Age, year, median (P25, P75)^*∗*^		33 (28, 40)	7.00 (3, 12)	—	8 (3, 12)	—	63 (52, 72)	—

Gender, *n* (%)	Female	3984 (96.51%)	7.00 (3, 12)	−2.349 (0.019)	8 (3, 12)	−1.070 (0.285)	63 (52, 72)	−0.566 (0.572)
	Male	144 (3.49%)	6 (1.25,1 1)		8 (2, 12)		62 (48, 72.75)	

Level of hospital, *n* (%)	Primary	405 (9.81%)	6 (2, 11)	9.822 (0.020)	6 (1, 10)	16.132 (0.001)	61 (48, 72)	60.403 (<0.001)
	Secondary	1150 (27.86%)	7 (3, 11)		8 (4, 12)		60 (50, 71.25)	
	Tertiary	2383 (57.73%)	7 (3, 12)		8 (3, 12)		65 (54, 72)	
	Unclassified†	190 (4.60%)	7 (2, 13)		7 (3, 14)		59 (48, 72)	

Type of hospital, *n* (%)	General hospital	3234 (78.34%)	7 (3, 12)	25.576 (<0.001)	8 (4, 12)	16.785 (<0.001)	64 (53, 72)	40.764 (<0.001)
	Specialized hospital	363 (8.79%)	7 (3, 11)		8 (4, 12)		61 (50, 71)	
	Community hospital	531 (12.86%)	6 (2, 11)		6 (2, 10)		59 (48, 72)	

Professional title, *n* (%)	Senior	462 (11.19%)	5 (2, 9)	52.582 (<0.001)	6 (1, 9)	46.827 (<0.001)	67 (56, 73)	33.981 (<0.001)
	Intermediate	1682 (40.75%)	7 (4, 13)		8 (4, 13)		64 (54, 72)	
	Junior and below	1984 (48.06%)	7 (3, 12)		8 (3, 12)		61 (49, 72)	

Marital status, *n* (%)	Unmarried	884 (21.41%)	6 (2, 10)	28.585 (<0.001)	7 (3, 11)	7.376 (0.061)	61 (49, 72)	17.317 (0.001)
	Married	3132 (75.87%)	7 (3, 12)		8 (3, 12)		64 (53, 72)	
	Divorced	100 (2.42%)	7 (2, 13.75)		8 (3, 14)		57 (48, 72)	
	Widowed	12 (0.29%)	5 (0.25, 14.25)		7 (0.75, 11)		60 (45.25, 72)	

Education, *n* (%)	College degree or below	1125 (27.25%)	6 (2, 10)	40.130 (<0.001)	7 (2, 11)	36.117 (<0.001)	62 (49, 72)	19.053 (<0.001)
	Bachelor's degree	2845 (68.92%)	7 (4, 13)		8 (4, 12)		64 (52, 72)	
	Master's degree	154 (3.73%)	6 (2, 9)		5 (1.75, 9)		68 (58, 73)	
	Doctor's degree or above	4 (0.10%)	4 (0.75, 6.50)		3 (0, 9)		63.5(61.50, 66.25)	

Vaccination, *n* (%)	No	343 (8.31%)	7 (3, 14)	−2.153 (0.031)	8 (4, 14)	−2.297 (0.022)	63 (51, 72)	0.316 (0.752)
	Yes	3785 (91.69%)	7 (3, 12)		8 (3, 12)		63 (52, 72)	

Family structure, *n* (%)	Live alone	659 (15.96%)	6 (2, 11)	72.808 (<0.001)	7 (2, 11)	37.079 (<0.001)	60 (48, 72)	24.246 (<0.001)
	Live with colleagues or friends	345 (8.36%)	7 (2.5, 11)		7 (3, 11)		62 (49, 72)	
	Husband and wife	595 (14.41%)	7 (3, 11)		7 (3, 11)		65 (53, 72)	
	Small family	1370 (33.19%)	7 (3, 12)		7 (3, 12)		65 (53, 72)	
	Three-generation family	1159 (28.08%)	7 (4, 14)		8 (4, 13)		62 (52, 72)	

Health status of parents, *n* (%)	Healthy	2050 (49.66%)	6 (2, 10)	106.364 (<0.001)	7 (2, 10)	89.595 (<0.001)	65 (52, 72)	26.643 (<0.001)
	One unhealthy	877 (21.25%)	7 (4, 13)		8 (4, 12)		61 (51, 72)	
	Both unhealthy	1201 (29.09%)	7 (4.5, 14)		9 (4, 14.5)		61 (52, 72)	

Number of children, *n* (%)	No child	1397 (33.84%)	6 (2, 10)	66.436 (<0.001)	7 (2.50, 11)	36.095 (<0.001)	63 (51, 72)	3.471 (0.325)
	One child	1750 (42.39%)	7 (3.75, 13)		8 (3, 12)		63 (53, 72)	
	Two children	958 (23.21%)	7 (4, 14)		9 (4, 13)		64 (51, 72)	
	Three children	23 (0.56%)	7 (4, 14)		8 (2, 11)		60 (48, 72)	

^*∗*^GAD, PHQ, PSSS scores, and age did not fit the normal distribution and were expressed as medians. ^†^Unclassified hospital: some small hospitals such as community health centers. ^§^Z: Mann–Whitney *U* test (for two-categorical variables); H: Kruskal–Wallis one-way ANOVA test (for k-categorical variables).

**Table 2:** Nucleic acid test results and vaccine completion status.

Nucleic acid test results	Total *n* = 4128	Vaccination^*∗*^		*χ* ^2^	*P* ^†^
		Yes	No		
COVID-19 positive with symptoms	1434 (34.74%)	1297 (31.42%)	137 (3.32%)	Ref-	Ref-
COVID-19 positive with no symptoms	98 (2.37%)	92 (2.23%)	6 (0.15%)	1.2761	0.2586
COVID-19 negative with symptoms	1179 (28.56%)	1097 (26.57%)	82 (1.99%)	5.6902	0.0171
COVID-19 negative with no symptoms	159 (3.85%)	155 (3.75%)	4 (0.10%)	8.7876	0.0030
No nucleic acid test with symptoms	1133 (27.45%)	1027 (24.88%)	106 (2.57%)	0.0290	0.8649
No nucleic acid test with no symptoms	125 (3.03%)	117 (27.99%)	8 (0.19%)	1.3556	0.2443

^*∗*^The full completion of vaccinations as required. ^†^The partitions of *χ*^2^ method were used. The control group consisted of nurses who tested positive for COVID-19 and reported symptoms. Test statistics values were considered statistically significant at *α*/2 (*k* − 1) = 0.005.

**Table 3:** Specific symptoms of nurses.

Symptoms	Total *n* = 4128
*Dyspnea*	
No	651 (10.93%)
Yes	3477 (89.07%)

*Chest distress*	
No	637 (15.43%)
Yes	3491 (84.57%)

*Fever (over 39°C)*	
No	978 (23.69%)
Yes	3150 (76.31%)

*Cough*	
No	1462 (35.42%)
Yes	2666 (64.58%)

*Headache*	
No	1654 (40.07%)
Yes	2474 (59.93%)

*Fatigue*	
No	1918 (46.46%)
Yes	2210 (53.54%)

**Table 4:** Generalized linear model of the factors associated with GAD scores (*n* = 4128).

Variable		Beta coefficient	Standard error	95% CI		Wald *χ*^2^	*p* value
				Lower	Upper		
Age		−0.007	0.0036	−0.014	0.000	3.582	0.058

Gender	Female	0.262	0.1659	−0.063	0.587	2.489	0.115
	Male	0^a^					

Level of hospital	Primary	−0.212	0.1666	−0.539	0.114	1.626	0.202
	Secondary	−0.235	0.1543	−0.538	0.067	2.325	0.127
	Tertiary	−0.154	0.1549	−0.457	0.150	0.985	0.321
	Unclassified^†^	0^a^					

Type of hospital	General hospital	0.440	0.1099	0.224	0.655	15.994	0.000
	Specialized hospital	0.142	0.1428	−0.138	0.422	0.988	0.320
	Community hospital	0^a^					

Professional title	Senior	−0.562	0.1190	−0.795	−0.328	22.266	0.000
	Intermediate	−0.060	0.0744	−0.205	0.086	0.642	0.423
	Junior and below	0^a^					

Marital status	Unmarried	−0.423	0.5862	−1.572	0.725	0.522	0.470
	Married	−0.166	0.5827	−1.309	0.976	0.082	0.775
	Divorced	−0.169	0.6069	−1.359	1.021	0.078	0.781
	Widowed	0^a^					

Education	College degree or below	1.152	0.9308	−0.672	2.977	1.533	0.216
	Bachelor's degree	1.355	0.9285	−0.465	3.174	2.128	0.145
	Master's degree	1.067	0.9398	−0.775	2.909	1.290	0.256
	Doctor's degree or above						

Vaccination	No	0.104	0.1073	−0.106	0.315	0.948	0.330
	Yes	0^a^					

Family structure	Live alone	−0.402	0.1190	−0.635	−0.169	11.401	0.001
	Live with colleagues or friends	−0.223	0.1046	−0.428	−0.018	4.552	0.033
	Husband and wife	−0.373	0.1373	−0.642	−0.104	7.394	0.007
	Small family	−0.227	0.0759	−0.375	−0.078	8.922	0.003
	Three-generation family	0^a^					

Isolation staff lounge preparation	No	−0.251	0.0650	−0.379	−0.124	14.961	0.000
	Yes	0^a^					

Traditional antiviral Chinese medicine preparation	No	0.125	0.0672	−0.007	0.257	3.459	0.063
	Yes	0^a^					

Ibuprofen preparation	No	0.255	0.0665	0.124	0.385	14.649	0.000
	Yes	0^a^					

Antitussive medicine preparation	No	0.062	0.0721	−0.079	0.204	0.748	0.387
	Yes	0^a^					

Acetaminophen preparation	No	0.010	0.0641	−0.116	0.136	0.025	0.875
	Yes	0^a^					

Health status of parents	Healthy	−0.593	0.0706	−0.732	−0.455	70.549	0.000
	One unhealthy	−0.267	0.0823	−0.428	−0.106	10.532	0.001
	Both unhealthy	0^a^					

Fever (over 39°C)	No	−0.238	0.0844	−0.403	−0.072	7.947	0.005
	Yes	0^a^					

Headache	No	−0.162	0.0981	−0.354	0.030	2.727	0.099
	Yes	0^a^					

Chest distress	No	−0.454	0.1131	−0.676	−0.232	16.116	0.000
	Yes	0^a^					

Dyspnea	No	−0.442	0.1102	−0.658	−0.226	16.053	0.000
	Yes	0^a^					

Cough	No	0.172	0.0837	0.008	0.336	4.225	0.040
	Yes	0^a^					

Fatigue	No	0.122	0.0982	−0.070	0.314	1.546	0.214
	Yes	0^a^					

Insufficient protective equipment	No	−0.295	0.0832	−0.458	−0.132	12.579	0.000
	Yes	0^a^					

Number of children		0.111	0.0539	0.006	0.217	4.262	0.039

PSSS family		−0.013	0.0094	−0.031	0.006	1.758	0.185

PSSS friend		−0.006	0.0112	−0.028	0.016	0.286	0.593

PSSS others		−0.067	0.0102	−0.087	−0.047	43.268	0.000

Dependent variable: GAD grade, none = 0, mild = 1, moderate = 2, severe anxiety = 3. ^a^Reference. ^†^Unclassified hospital: some small hospitals such as community health centers. ^*∗*^Type of family structure: small family: couple and children living together. Three-generation family: couple, children, and grandparents living together.

## Data Availability

Data that support the findings of this study are available upon reasonable request. If necessary, please contact email 382731326@qq.com.

## Additional Points

*What Is Already Known*. (i) Frontline nurses experienced significant psychological stress during the COVID-19 epidemic. (ii) After the Chinese government dropped its “dynamic zero strategy,” a large number of people were infected, creating a peak period of the epidemic. (iii) Overloaded work schedules and insufficient sleep became a common situation during COVID-19 peak infection period. *What This Paper Adds*. (i) Nurses' anxiety levels were even worse during the peak of the epidemic. (ii) Worries about family members became the main concern of the nurses. (iii) Nurses were worried about complaints and expected understanding from the public during the peak of the epidemic.
